# Incidence of periprosthetic joint infection after primary total knee arthroplasty shows significant variation : a synthesis of meta-analysis and bibliometric analysis

**DOI:** 10.1186/s13018-024-05099-8

**Published:** 2024-10-12

**Authors:** Tao Ma, Jun Jiao, Da-Wei Guo, Shu-Zheng Lv, Di Zhang, De-Cai Hou

**Affiliations:** 1 Shenyang Orthopedic Hospital, Shenyang, China; 2 Affiliated Hospital of Liaoning University of Traditional Chinese Medicine, Fuxin, China

**Keywords:** Periprosthetic joint infection, Total knee arthroplasty, Incidence, Epidemiology, Meta-analysis, Bibliometric analysis

## Abstract

Total Knee Arthroplasty (TKA) is a surgery that is commonly performed on older adults to improve their quality of life. However, the increasing use of knee joint prostheses has led to a rise in the incidence of Prosthetic Joint Infections (PJI) in patients after TKA. Different clinical studies have looked at the occurrence of PJI after TKA in different regions, but they have drawn varying conclusions. To better understand this topic, we conducted a meta-analysis and bibliometric study using data from multiple databases. Our research found that the estimated prevalence of PJI after TKA is approximately 1.08% across different regions, but there is still considerable variation. Additionally, our regression analysis of sub-groups shows significant differences in follow-up periods. Furthermore, our comprehensive bibliometric analysis identifies current research trends, “hotspots” related to TKA-related PJI, influential nations, organizations, and noteworthy publications. Our analysis provides valuable insights to guide future research in this area.

**Supplementary Information:**

The online version contains supplementary material available at 10.1186/s13018-024-05099-8.

## Introduction

Total knee arthroplasty (TKA) is a well-established, safe, and effective procedure, widely performed in orthopedics to enhance quality of life and restore function in patients with knee arthritis [1, 2]. The growing number of knee prostheses being implanted is largely attributed to the increasing life expectancy of the elderly population and their heightened demand for an improved quality of life [3, 4]. About 1 million knee and hip replacements are performed in the U.S. each year, and that number is expected to double by 2030 [5]. In China, with nearly 110 million knee osteoarthritis patients in 2016, the potential patient population in need of TKA is huge [6]. The dramatic increase in surgical volume poses unprecedented challenges to perioperative management and postoperative rehabilitation.

The reasons for TKA surgical failure requiring revision are complex and varied and include PJI, instability, component misalignment, polyethylene or metal wear, loose cement, periprosthetic fracture, instability, or knee stiffness [7, 8]. PJI is a major contributor to TKA failure and the incidence is on the rise [9]. Although the incidence of PJI reported after TKA is in the range of 0.5-2%, the overall global burden is substantial [10]. Once PJI occurs, its consequences can be catastrophic, causing significant impacts on society, families, and individuals. Risk factors for developing PJI after TKA have been reported and include hypertension, diabetes mellitus, hyperlipidemia, nephropathy, urinary tract infection, overweight, wound complications, and poor nutritional support [11, 12]. In conclusion, diagnosing and treating PJI is particularly challenging in orthopedic practice. The complexity arises from the difficulty in accurately identifying the infection, coupled with the need for tailored treatment strategies that address both infection eradication and complications. In 2018, new diagnostic criteria for PJI were introduced internationally, improving the sensitivity and specificity of PJI diagnosis [13, 14]. The Musculoskeletal Infection Society defines the major criteria for diagnosing PJI as either the presence of two positive cultures of the same organism obtained through standard culture methods or the presence of a sinus tract with evidence of communication to the joint or direct visualization of the prosthesis. Additionally, minor criteria include several indicators such as elevated C-reactive protein, elevated D-dimer levels, and elevated erythrocyte sedimentation rate, among others, which can also support the diagnosis of PJI [13]. However, numerous clinical studies have often been inconsistent in their results, with significant variations. Therefore, more comprehensive identification of potential risk factors and the incidence of PJI is critical.

In this study, we used meta-analysis combined with bibliometrics to investigate the prevalence and potential risk factors of PJI. Meta-analysis and bibliometrics can altogether help us to systematically analyze and combine data from multiple studies, comprehensively assess the value of literature, and provide strong data support for our research [15, 16].

## Method

### Meta-analysis

This is a systematic review registered with the Prospero repository under the ID CRD42024527125. The search for relevant studies began at the start of the project and continued until March 2024. The search included three major medical repositories: PubMed, Embase, and Web of Science. The search terms used were “infection or infections or PJI”, “prevalence or incidence or epidemiology”, and “TKA or knee or arthroplasty”, combined with “periprosthetic or implant or prosthesis or prosthesis-related or periprosthesis-related”. There were no language restrictions during the search, and the final search was conducted on March 15, 2024.

### Inclusion and exclusion criteria

Inclusion criteria: patients of any age who have undergone primary total knee arthroplasty (TKA); no restrictions on publication date; studies with large sample sizes were preferred; and the research should report the incidence of prosthetic joint infection (PJI) after primary TKA. Exclusion criteria: reviews, case studies, protocols, abstracts, personal commentaries, letters, posters, conference abstracts or laboratory-based investigations; studies without sufficient data or not accessible; and research not related to PJI patients.

### Data extraction

Evaluation of study articles was based on their titles and abstracts. Abstracts and any articles without abstracts were chosen for a full-text assessment. Two researchers independently extracted data from the selected studies and cross-verified the findings to maintain the accuracy of the information. In instances of discrepancies, a third researcher was brought in to resolve the discrepancy(Table 1**)**.

**Table 1:** Characteristics of the database-based studies of PJI

Study	Country	PJI	Primary TKA	Male PJI	Male TKA	Female PJI	Female TKA	Year	Age	Follow-up time	Data Sources	Publication Year
Yoon H.K. et al. 2023	Korea	34	7019	5	/	29	/	2003–2019	69.2	> 12 m	Single institution	2023
Ashkenazi I. et al. 2023	Israel	7	1590	/	/	/	/	2011.1-2021.4	69.9(± 9.8)	/	Single institution	2023
Hameed D. et al. 2023	USA	81	5000	/	/	/	/	2015–2020	66	12–24 m	Database	2023
Stevoska S. et al. 2022	Australia	24	2666	/	1011	/	1655	2011–2020	/	/	Single institution	2022
Kurtz S.M. et al. 2022	USA	619	89,837	/	/	/	/	2010–2017	> 65	0–24 m	Database	2022
Hasenauer M.D. et al. 2022	USA	105	8462	56	3176	49	5466	2006–2018	66.9(33–95)	/	Single institution	2022
Bozzo A. et al.2022	Canada	1834	129,613	/	50,453	/	79,160	2002–2016	> 50	/	Database	2022
Yang Q.F. et al. 2021	China	2455	1,227,244	/	/	/	/	2005–2014	> 18	/	Database	2021
Baier C. et al. 2019	Germany	75	2439	/	/	/	/	2007–2010	/	/	Single institution	2019
Siu K.T. et al. 2018	China(Hong Kong)	34	2543	10	539	24	2004	1993–2013	69(21–91)	/	Single institution	2018
Poultsides L.A. et al. 2018	USA	47	17,959	/	6525	/	11,434	2000–2009	68.15(12.6–96.8)	/	Database	2018
Klement M.R. et al. 2016	USA	7629	1,271,464	/	39,355	/	154,749	2005–2011	/	/	Database	2016
Alp E. et al. 2016	Turkey	11	241	/	/	/	/	2011.4-2013.4	64	/	Single institution	2016
Lee Q.J. et al. 2015	Hongkong	8	1133	4	/	4	/	2011–2014	/	/	Single institution	2015
Jämsen E. et al. 2010	Finland	24	2647	12	784	12	1863	2002–2006	70 (35–97)	> 6 m	Single institution	2010
Pasticci M.B. et al. 2007	Italy	3	171	/	62	/	109	1997–2002	71(37–82)	> 24 m	Single institution	2007
Cook J.L. et al. 2007	USA	15	3013	7	/	8	/	1985–2004	67(40–79)	/	Single institution	2007
Wang FD et al. 2018	China(Taiwan)	178	10,768	/	3527	/	7241	2002–2014	> 18(中位数73)	0–36 m	Single institution	2018
Ko MS et al. 2021	Korea	12,474	560,954	/	/	/	/	2005–2018	/	/	Database	2021
Jung P et al. 2017	New Zealand	44	9481	31	4348	13	5085	2013–2015	70	0-90d	Database	2017
Phillips JE et al. 2006	England	41	4788	/	/	/	/	1987–2001	67.6(49–85)	13.8 m	Single institution	2006
Keemu H. et al. 2023	Finland	484	62,087	/	22,941	/	39,146	2014–2020	/	/	Database	2023
Bourget Murray J. et al. 2023	Canada	275	39,038	/	/	/	/	2013.1-2020.3	/	0–3 m	Database	2023
Jin X. et al. 2022	Australia	192	191,914					2002.1-2017.12	/	0–3 m	Database	2022
Teo B.J.X. et al. 2018	Singapore	4	905	1	194	3	711	2004–2014	65.9 ± 7.7	/	Single institution	2018
Koh C.K. et al. 2017	New Zealand	169	11,134					2000–2015	69 (9.7)	0–180 m	Single institution	2017
Fan J.C.H. et al. 2008	China(Hong Kong)	5	479	1	83	4	396	1997–2006	69(40–88)	46(1-107)m	Single institution	2008

### Quality assessment

We used the JBI Quality Appraisal Tool for Systematic Reviews and Meta-Analyses to evaluate article quality. This tool examines study selection, data compilation, and presentation of findings, allowing readers to gauge a study’s reliability and relevance. It’s a critical instrument by the Australian-based Joanna Briggs Institute. For detailed scores, see Additional file 1: Table 1. Higher scores mean better research quality and less bias. We ranked studies into High (scores below 49%), medium (scores between 50 and 69%), and low (scores exceeding 70%) quality categories.

### Data analysis

The study’s data was analyzed using the Meta module in R version 4.0.5. We converted incidence rates using the logarithm method and tested normality using the Shapiro-Wilk test. We used Q and I^2^ statistics to assess heterogeneity, with *P* < 0.05 and I^2^ > 50% indicating statistical heterogeneity [15]. In general, when *P* > 0.05 (for Q statistic) and I^2^ < 50%, the combined result is statistically homogeneous and a fixed-effects model can be used; when *P* > 0.05 and I^2^ > 50%, it indicates statistical heterogeneity and a random-effects model should be used. We used a forest plot to display incidence rates and the Egger test to assess publication bias. Meta-regression analysis was used to examine heterogeneity. Subgroup analyses were conducted by publication time, geographic location, gender, search standards, age range, and post-THA PJI incidence time.

### Bibliometrics analysis

#### Data acquisition and search strategy

For this study, we relied on the Web of Science Core Collection (WOSCC) as our principal database for gathering information. On March 12, 2024, we executed a search according to a predetermined protocol: (TS = (total knee arthroplasty)) AND TS = (infection). We did not limit our search to any specific language. To maintain the integrity and pertinence of the selected articles, we excluded various forms of content, including editorials, letters, conference abstracts, revisions, conference proceeding papers, book sections, and retracted articles.

### Data analysis

Data was gathered from publications in the Web of Science Core Collection and analyzed using two specialized bibliometric tools: R version 4.2.0 and VOSviewer. The R-based package Bibliometrix was used to process the data set, which enabled the creation of a range of bibliometric indicators and visual outputs. This comprehensive toolset supports the overall processes of data importation, management, thorough analysis, and visual display of bibliometric information.

VOSviewer is a web and knowledge graph visualization software for developing scientometrics [17]. VOSviewer can be used to identify highly effective journals and co-cited journals Importing the research data into VOSviewer for visualization allows us to analyze different elements, including countries, journals, and keywords. The research data was imported into VOSviewer for analysis, the appropriate parameters were set, and then visualizations including citations, bibliographic coupling, co-citations, or co-authors were performed.In the visual maps created by VOSviewer, each point is represented by a circular icon with a label indicating its identity. Co-occurrence analysis reveals that circles with greater size signify higher frequencies of occurrence. The color of these circular elements is determined by the cluster to which they belong. The thickness of the lines connecting the nodes reflects the intensity and significance of the relationship and the correlation between specific nodes.

## Result

### Meta-analysis

#### Literature search and included studies

**Fig. 1:**
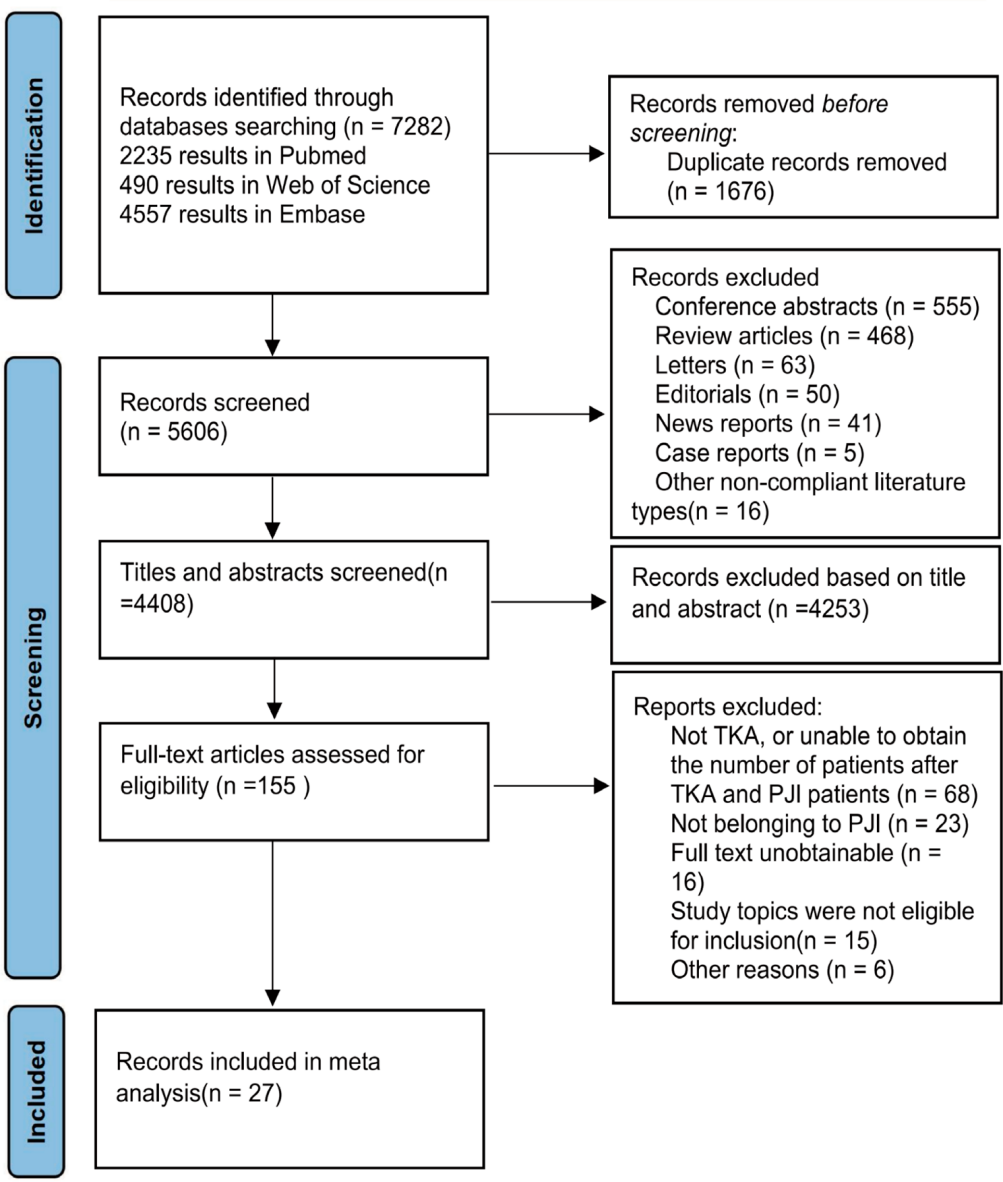
Candidate study selection workflow for metaanalysis

Using the search term “Arthroplasty, Replacement, Knee“[Mesh] OR “Arthroplasties, Replacement, Knee” OR “Arthroplasty, Knee Replacement” OR “Knee Replacement Arthroplasties” OR “Knee Replacement Arthroplasty” OR “Replacement Arthroplasties, Knee” OR “Knee Arthroplasty, Total” OR “Arthroplasty, Total Knee” OR “Total Knee Arthroplasty” OR “Replacement, Total Knee” OR “Total Knee Replacement” OR “Knee Replacement, Total” OR “Knee Arthroplasty” OR “Arthroplasty, Knee” OR “Arthroplasties, Knee Replacement” OR “Replacement Arthroplasty, Knee” resulted in 7282 records from the 3 databases (2235 results in Pubmed; 90 results in Web of Science; 4557 results in Embase). After duplicate removal, our literature searches yielded 5606 articles. We screened 4408 potentially relevant reports, reviewed 155 articles in full-text, and fnally included 27 studies in the analysis. Details of the inclusion process are shown in Fig. 1. The characteristics of studies are summarized in Table 1. The 27 studies, with a combined population of 3,664,589, cover 4 continents (America, Oceania, Europe, and Asia) and 13 countries (including the USA, Australia, China, Korea, Finland, Canada, New Zealand, England, Singapore, Italy, Turkey, Germany and Israel). The JBI quality assessment showed that 11 studies had a high risk bias, 10 studies had a moderate risk bias, and 6 studies had a low risk bias. The whole process was done by two researchers. A third researcher decides when disagreements arise.

### Overall incidence of PJI

**Fig. 2:**
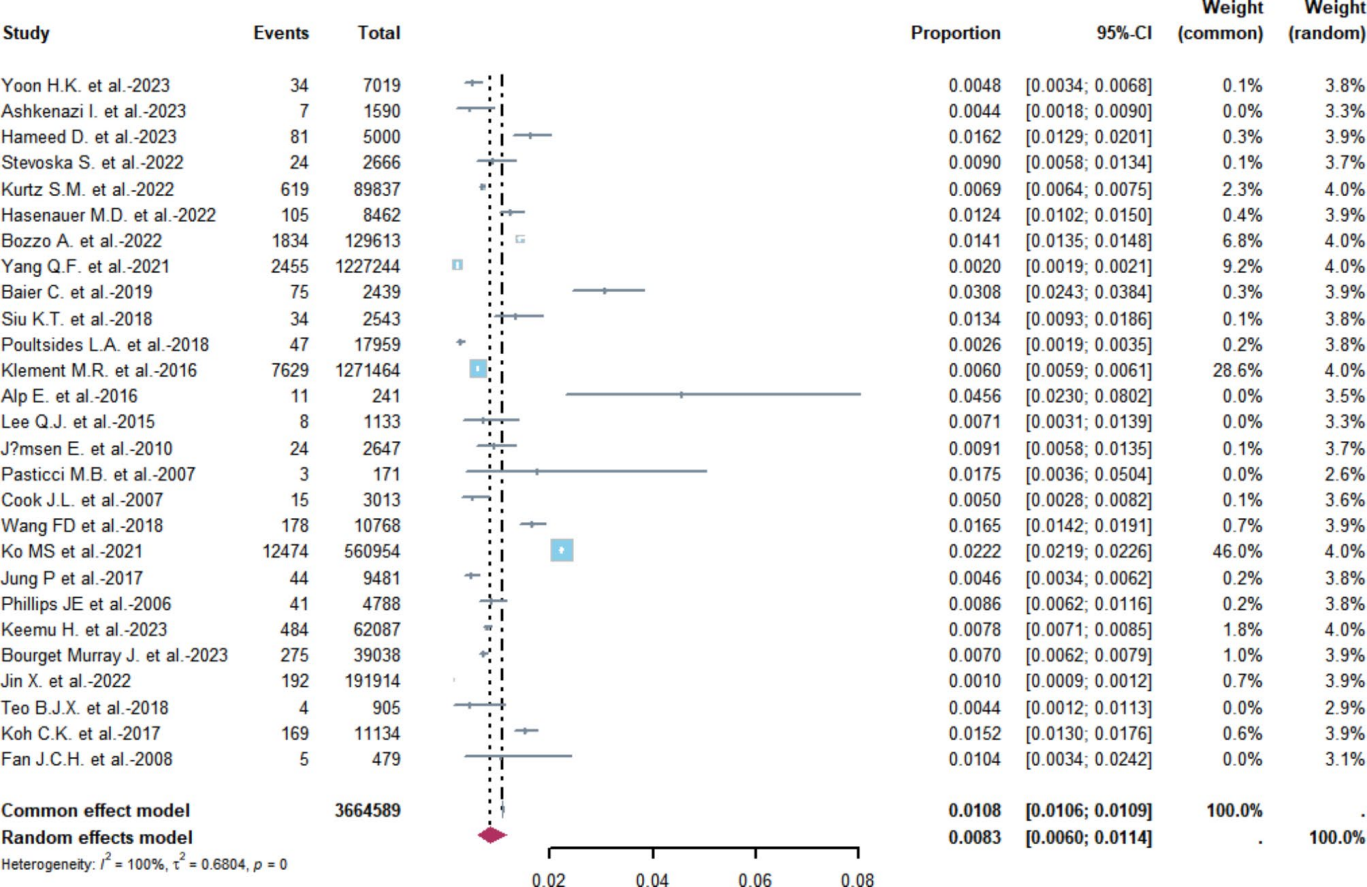
Forest plot of the overall incidence of PJI in the database-based studies. The size of the squares represents the proportion (95% CI) for each of the studies. The size of the diamonds represent the overall proportion (95% CI)

Based on the figure presented, a meta-analysis was conducted to estimate the incidence rates of PJI. The combined fixed-effects model estimate of the overall PJI incidence rate was found to be 1.08% (95% CI 1.06–1.09%). Meanwhile, the overall combined random-effects estimate was 0.83% (95% CI 0.6–1.14%). It is important to note that the heterogeneity was found to be extremely high (I^2^ = 100%; Heterogeneity test *P* = 0), we therefore adopted the results of the random effects(Fig. 2).

### Incidence of PJI by publication time

According to the literature included in this study, the publication time of papers was divided into two categories: those published within the last ten years and those published more than ten years ago. The purpose of categorization was to estimate the incidence rate of PJI. The study included 22 papers published between 2014 and 2023. The fixed effects overall combined estimate of PJI occurrence rate was 1.08% (95% CI 1.06–1.09%), while the random effects overall combined estimate was 0.83% (95%CI 0.6–1.14%). Since I^2^ > 50%, we adopted the results of the random effects here. The high degree of heterogeneity (I^2^ = 100%; Heterogeneity test *P* = 0) suggested significant variation among the included papers. Before 2013, the study included five papers, and the fixed effects overall combined estimate of PJI occurrence rate was 0.82% (95% CI 0.67%-1.01%). The random effects overall combined estimate was 0.82% (95%CI 0.63-1.07%), which also showed low heterogeneity (I2 = 35%; Heterogeneity test *P* = 0.19) (Supplementary material: Fig. 1).

### Incidence of PJI by sex

Several studies have shown that there are gender differences in the incidence rate of PJI. For males, the overall estimate of the incidence rate for PJI was 1.33% (95% CI 1.11–1.6%) under a combined fixed-effect, and 1.29% (95% CI 0.84–1.97%) under a random-effects model, indicating no high heterogeneity (I^2^ = 74%; Heterogeneity test *P* < 0.01). For females, the overall estimate of the incidence rate for PJI was 0.78% (95% CI 0.64–0.94%) in a fixed-effects model, and 0.67% (95% CI 0.41–1.10%) in a random-effects model, with less heterogeneity observed (I^2^ = 79%; Heterogeneity test *P* < 0.01) (Supplementary material: Fig. 2).

### Incidence of PJI by geographic location of conducted studies

Our study analyzed the incidence rate of PJI (Periprosthetic Joint Infection) based on the geographic location of conducted studies. The studies included four major continents: Asia, North America, Oceania, and Europe. In Asia, we found that the fixed effect summary estimate of PJI incidence rate was 1.49% (95% CI 1.47–1.51%), whereas the random effect summary estimate was 0.90% (95% CI 0.50–1.63%). The heterogeneity was extreme (I^2^ = 100%; Heterogeneity test *P* < 0.01). In North America, the fixed and random effects summary estimates of PJI incidence were 0.71% (95% CI 0.70–0.73%) and 0.77% (95% CI 0.50–1.16%), respectively. The heterogeneity was high (I^2^ = 99%; Heterogeneity test *P* < 0.01). For Oceania, the fixed effect summary estimate for PJI was 0.38% (95% CI 0.35–0.42%), and the random effect summary estimate was 0.50% (95% CI 0.16–1.60%). We observed extreme heterogeneity (I^2^ = 100%; Heterogeneity test *P* < 0.01). Finally, in Europe, the fixed effect summary estimate of PJI incidence was 0.93% (95% CI 0.86–1.01%), and the random effect summary estimate was 1.23% (95% CI 0.70–2.16%). We found extreme heterogeneity (I^2^ = 97%; Heterogeneity test *P* < 0.01) in this continent as well (Supplementary material: Fig. 3). The latter result is therefore acceptable.

### Incidence of PJI by follow-up time

It has been reported in previous studies that PJI can occur up to one year after TKA. Therefore, we divided our study into two groups: those within a year and those over a year and conducted a subgroup analysis based on follow-up time. For studies that followed up within a year, the overall fixed effect estimation of PJI occurrence rate was 0.95% (95% CI 0.90–1.01%), and the overall random effect estimation of occurrence rate was 1.11% (95% CI 0.74–1.66%), showing high heterogeneity (I^2^ = 97%; heterogeneity test *P* < 0.01). For studies with follow-up times exceeding one year, the fixed effect institution’s overall merged estimated rate of PJI occurrence was 0.35% (95% CI 0.32–0.38%), and the random effect institution’s overall merged estimated rate of PJI occurrence was 0.41% (95% CI 0.16–1.06%), again showing high heterogeneity (I^2^ = 99%; Heterogeneity test *P* < 0.01).

### Publication bias

We performed an analysis to detect publication bias in the studies that we included. The funnel plot (Supplementary material: Fig. 3) shows some asymmetry on both sides of the symmetry axis among the studies. However, we also conducted the Egger test (Supplementary material: Fig. 6) and found that there is no statistically significant publication bias (*P* > 0.05).

### Sensitivity analyses

We carefully examined and excluded each piece of literature that we included in our analysis. We also looked at how much influence each individual study had on the overall effect size. Our findings showed that there was no significant change in the effect size, which indicates that the included studies were stable and reliable (Supplementary material: Fig. 6).

### Meta-regression

**Fig. 3:**
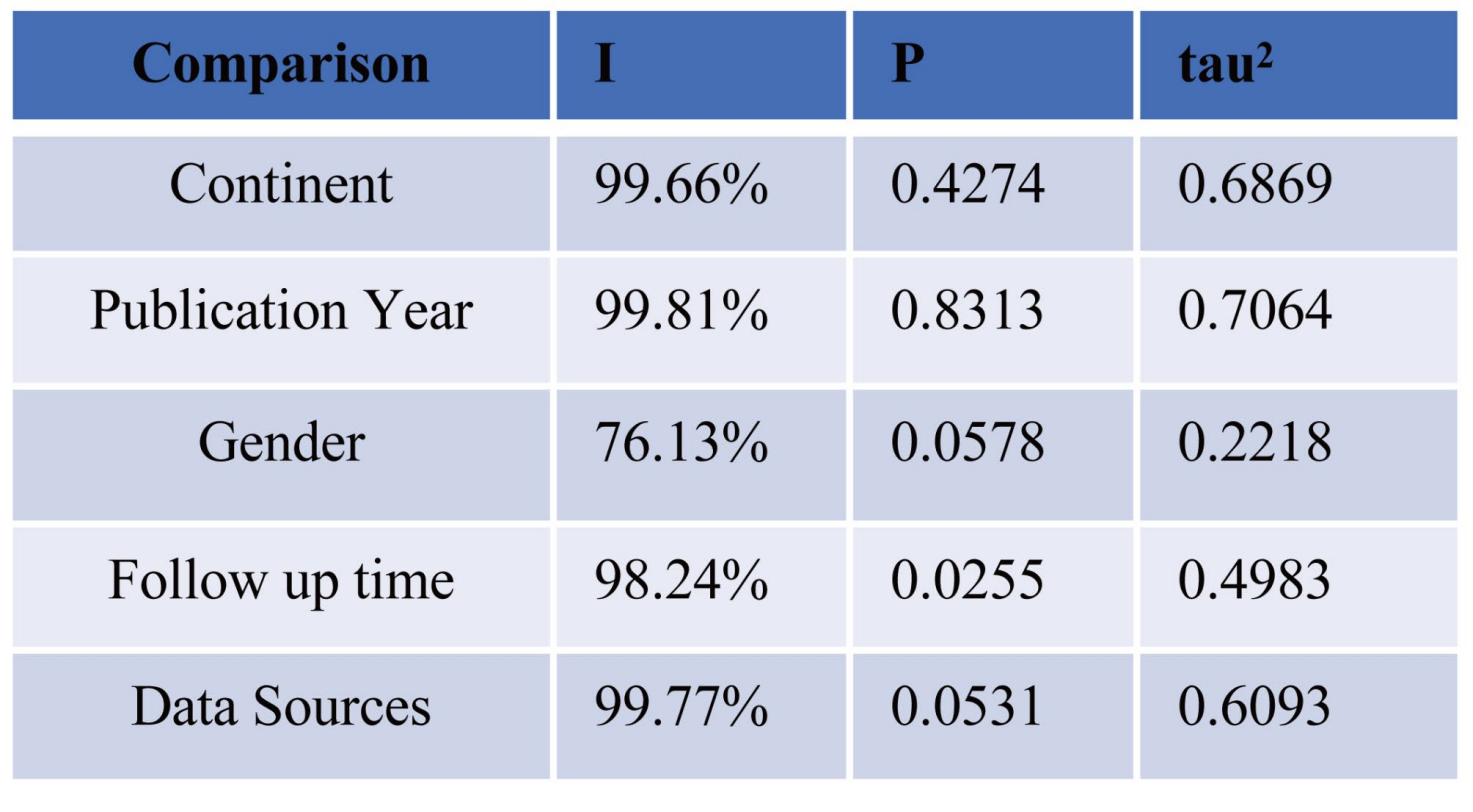
The Meta-regression analysis

We conducted a Meta univariate regression analysis on subgroups to address the issue of high heterogeneity. The results indicate that, in database-based research, the follow-up time is the source of heterogeneity (*P* < 0.05), while the other factors are not *(P* > 0.05) (Fig. 3).

### Incidence of PJI by Data sources

**Fig. 4:**
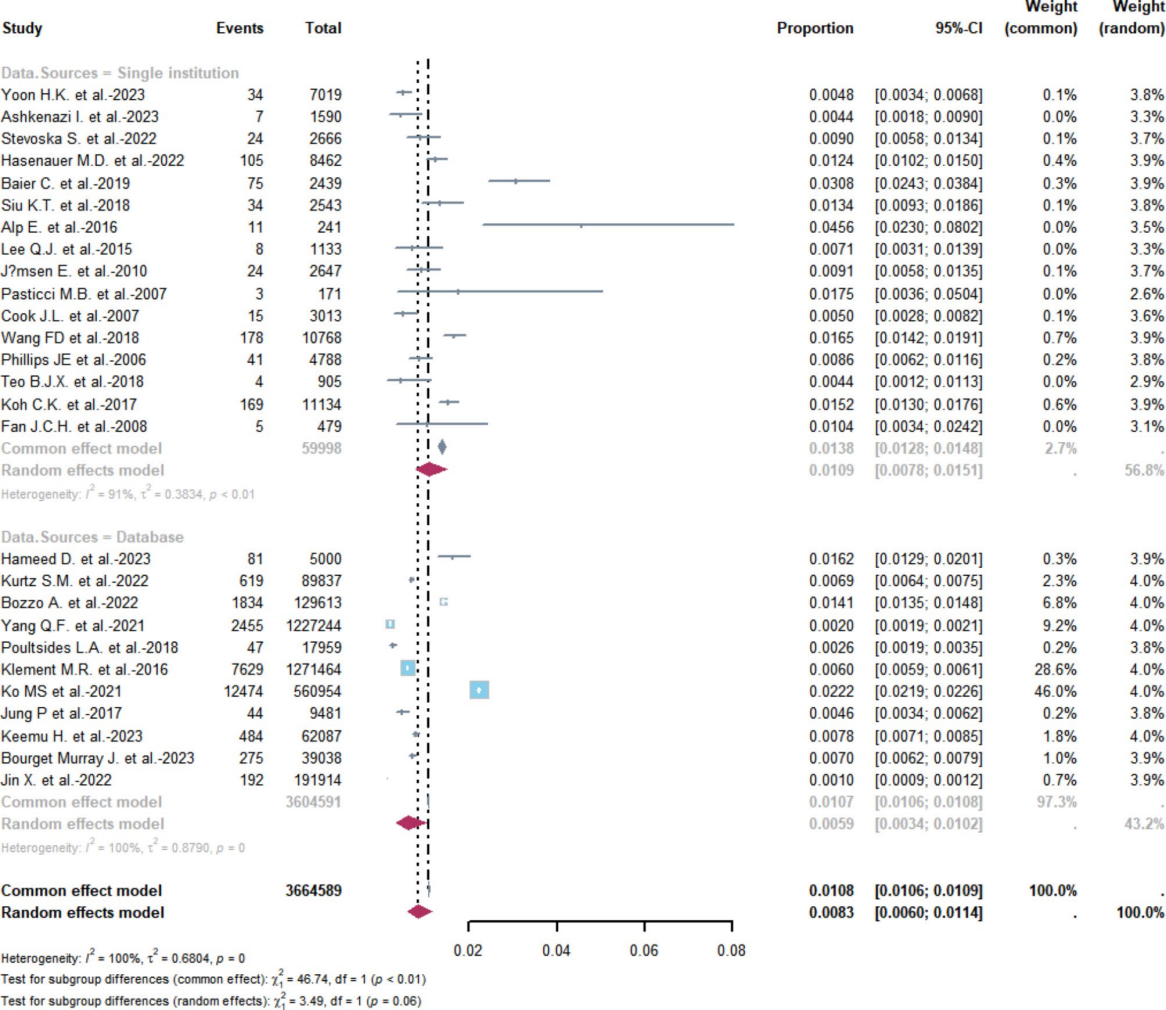
Incidence of PJI from a single institution or database

In the single-institution studies, a total of 16 research papers were analyzed. As shown in Fig. 4 The overall combined occurrence rate of PJI was estimated to be 1.38% (95% CI 1.28–1.48%) with a fixed effect, while the total combined occurrence rate was estimated to be 1.09% (95% CI 0.78–1.51%) with a random effect. However, there was a high level of heterogeneity (I^2^ = 91%; heterogeneity test *P* < 0.01). In the case of database studies, a total of 11 papers were analyzed. The overall combined occurrence rate of PJI was estimated to be 1.07% (95% CI 1.06–1.08%) with a fixed effect, while the overall combined occurrence rate was estimated to be 0.59% (95% CI 0.34–1.02%) with a random effect. The level of heterogeneity was extremely high (I^2^ = 100%; heterogeneity test *P* = 0). We therefore choose the latter one.

### Bibliometric analysis

#### Basic data summary

In this study, we primarily utilized the Web of Science Core Collection (WOSCC) database as our principal information source. On March 14, 2024, a predefined search strategy was employed: (TS=(total knee arthroplasty)) AND TS=(infection). No language restrictions were enforced on the publications. To ensure quality and relevance, several types of materials, such as editorials, letters, conference abstracts, revisions, proceedings, book chapters, and retracted publications, were excluded.

A total of 3,831 articles were subsequently analysed in this study. Authored by 12,715 researchers from 3,670 institutions across 70 countries since 1994, these papers were published across 442 different journals. Each article, on average, has been cited 28.48 times. According to Lotka’s Law, the authors that contribute to a research study (1.2) make up 50% of the total number of authors.

#### Annual number of publications

**Fig. 5:**
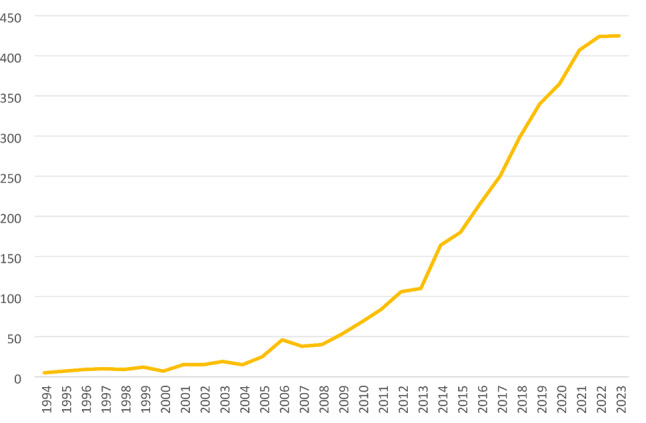
Annual publication numbers from 1994 to 2023

Since 2009, there has been a notable surge in the volume of scholarly articles published on PJI following primary TKA. The growth rate in that year was 32.5%, and subsequently, up until the end of 2023, research institutions globally have averaged yearly contributions of 233 articles in this field (Fig. 5 ) (Supplementary materiale: Table 2).

#### Analysis of the countries with the highest productivity

The greatest volume of publications comes from the USA (*n* = 1557), followed by Germany (*n* = 522), China (*n* = 338), England (*n* = 228), Italy (*n* = 184), Spain (*n* = 165), France (*n* = 160), and Switzerland (*n* = 108). All other countries/regions have published fewer than 100 articles each. Most research collaborations involve the USA and other countries, with the top four being between the USA and China (*n* = 43), USA and Israel (*n* = 39), USA and England (*n* = 32), and USA and Germany (*n* = 32).

#### Analysis of the most productive journals

The top ten journals in publication volume, as presented above, have contributed to 44.17% (1692 out of 3831) of the total articles in this field. Three of them are based in the United States, another three in the United Kingdom, and the remaining three in Germany. They are primarily from the sphere of Orthopedics, however, some represent other fields such as Surgery, Rheumatology, and Sports Sciences (Table 2).

**Table 2:** The table of most productive journals

Journal	Articles	IF(2023)	field/JCR
JOURNAL OF ARTHROPLASTY	784	3.5	ORTHOPEDICS (Q1)
CLINICAL ORTHOPAEDICS AND RELATED RESEARCH	189	4.2	ORTHOPEDICS(Q1)/SURGERY (Q1)
JOURNAL OF BONE AND JOINT SURGERY AMERICAN VOLUME	157	5.3	ORTHOPEDICS(Q1)/SURGERY (Q1)
BONE … JOINT JOURNAL	111	4.6	ORTHOPEDICS(Q1)/SURGERY (Q1)
ARCHIVES OF ORTHOPAEDIC AND TRAUMA SURGERY	108	2.3	ORTHOPEDICS(Q2)/SURGERY (Q2)
INTERNATIONAL ORTHOPAEDICS	95	2.7	ORTHOPEDICS(Q2)
BMC MUSCULOSKELETAL DISORDERS	64	2.3	ORTHOPEDICS(Q3)/RHEUMATOLOGY(Q4)
KNEE SURGERY SPORTS TRAUMATOLOGY ARTHROSCOPY	62	3.8	ORTHOPEDICS(Q1)/SPORT SCIENCES(Q1)/SURGERY(Q1)
JOURNAL OF KNEE SURGERY	61	1.7	ORTHOPEDICS(Q3)
JOURNAL OF ORTHOPAEDIC SURGERY AND RESEARCH	61	2.6	ORTHOPEDICS (Q2)

#### Analysis of the most cited publications

Table 3 shows that the top ten most-cited research papers in this field were published around 2010. The most frequently cited paper is “ZIMMERLI W, 2004”, which provides a comprehensive overview of the pathogenesis, diagnosis, and treatment of PJI.

**Table 3:** The top 10 most cited publications

Paper	DOI	Total Citations	TC per Year	Journal	IF	JCR
ZIMMERLI W, 2004, NEW ENGL J MED	10.1056/NEJMra040181	2114	100.67	The New England Journal of Medicine	158.5	Q1
OSMON DR, 2013, CLIN INFECT DIS	10.1093/cid/cis803	1673	139.42	Clinical Infectious Diseases	11.8	Q1
KURTZ SM, 2012, J ARTHROPLASTY	10.1016/j.arth.2012.02.022	1190	91.54	The Journal of arthroplasty	3.5	Q1
TANDE AJ, 2014, CLIN MICROBIOL REV	10.1128/CMR.00111 − 13	1058	96.18	Clinical Microbiology Reviews	36.8	Q1
PARVIZI J, 2018, J ARTHROPLASTY	10.1016/j.arth.2018.02.078	1016	145.14	The Journal of arthroplasty	3.5	Q1
TRAMPUZ A, 2007, NEW ENGL J MED	10.1056/NEJMoa061588	948	52.67	The New England Journal of Medicine	158.5	Q1
PULIDO L, 2008, CLIN ORTHOP RELAT R	10.1007/s11999-008-0209-4	915	53.82	Clinical Orthopaedics and Related Research	4.2	Q1
PARVIZI J, 2011, CLIN ORTHOP RELAT R	10.1007/s11999-011-1971-2	836	59.71	Clinical Orthopaedics and Related Research	4.2	Q1
KURTZ SM, 2008, J ARTHROPLASTY	10.1016/j.arth.2007.10.017	717	42.18	The Journal of arthroplasty	3.5	Q1
DEL POZO JL, 2009, NEW ENGL J MED	10.1056/NEJMcp0905029	575	35.94	The New England Journal of Medicine	158.5	Q1

#### Analysis of keywords

After consolidating keywords with the same meaning, our search yielded a total of 7519 author keywords. We then filtered out keywords that occurred less than 20 times, resulting in 249 keywords that met our filtering criteria. Figure 2 displays the frequency of these keywords; the larger the yellow circle, the higher the keyword’s occurrence.

**Fig. 6:**
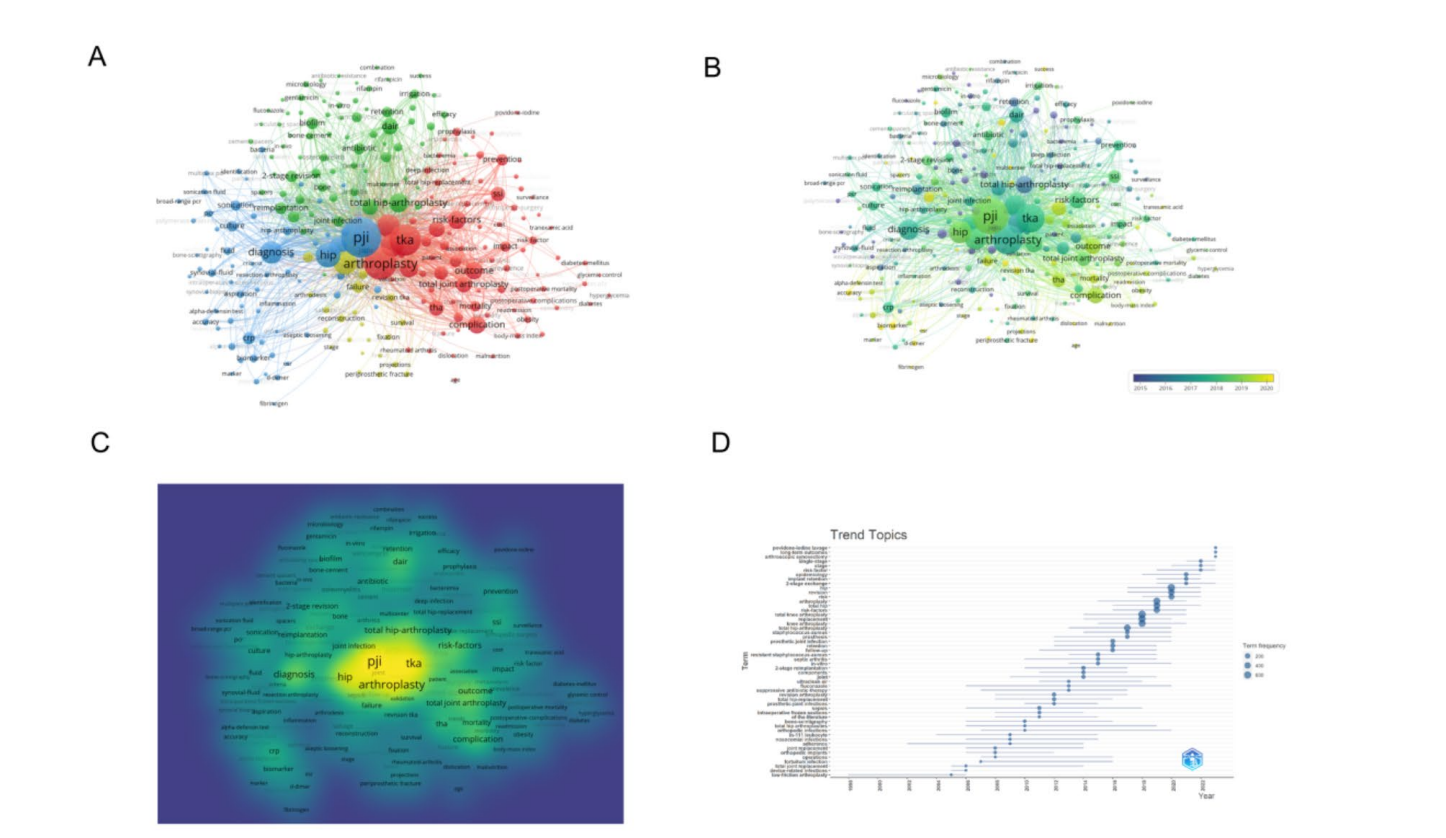
**A** network visualization of author keywords; **B** visualisation map summarising the themes of the previous years from a temporal point of view; **C** Visualisation maps of different research branch networks; **D** Popular Hot Topic Words of the Past Years

Figure 6A provides a visual analysis of the relationships between these high-frequency keywords. The size of each node corresponds to the frequency of the keyword, while the distance between two nodes indicates their degree of correlation. Keywords located closely together are grouped into the same cluster (denoted by color), reflecting the central research themes of that specific branch. The keyword analysis resulted in four clusters. Cluster 1, highlighted in red, includes high-frequency words reflecting the prevention of PJI, such as risk-factors, outcome, complication, risk, and prevention. Cluster 2, colored in green, contains keywords like dair, management, reimplantation, biofilm, 2-stage revision, antibiotic, two-stage revision, indicative of PJI treatment. Cluster 3, marked in blue, features keywords such as diagnosis, crp, culture, sonication, septic arthritis, fluid, which centers on PJI diagnosis. Lastly, Cluster 4, denoted in yellow, represents the management of initial TKA and revision failure with keywords such as revision, failure, prosthesis, follow-up, revision tka, reconstruction, salvage, survival.

Research topics are not stagnant; a chronological assessment of keywords from previous years can reveal the shift of research focus in this field. In Fig. 6B, darker colors indicate earlier research periods, and lighter colors represent more recent studies. Combined with the viewpoint of different research branches displayed in Fig. 6C, it’s noticeable that until 2018, PJI diagnosis and treatment were the focal points of research. From 2018, researchers gravitated towards PJI prevention and management post-occurrence. Figure 6D demonstrates these shifting research trends from the perspective of popular keywords over the years. The notable keywords for the recent two years in the field include povidone-iodine lavage, long-term outcomes, arthroscopic synovectom.

## Discussion

### Meta-analysis

Our research has found that the total incidence rate of PJI stands at 0.83%. However, when we analyzed different sources of data, we found a significant difference in the incidence of PJI between database sources and individual institutions. The former showed an incidence of only 1.09%, while the latter recorded an incidence rate of 0.59%. This difference may be due to several factors, including the vast population data available in the databases compared to smaller sample sizes in individual institutions. The incidence of PJI after TKA in the general population ranges from 0.5 to 2.3% in USA, which is different from our results [18]. We believe that this difference may be due to the larger number of TKA recipients in the United States. The treatment of TKA presents a significant challenge due to numerous factors influencing the incidence of PJI. This is a research area that is being actively pursued by many scholars.

In 2013, the International Consensus Meeting (ICM) introduced new diagnostic criteria for PJI, which resolved previous confusion about its diagnosis. As a result, we analyzed the data before and after 2014, the year the new criteria were established. The analysis showed that the incidence rate of PJI was lower (0.82%) before 2014 compared to the period after 2014 (1.08%). This suggests that the introduction of new diagnostic criteria has led to better detection of PJI, which was previously overlooked. The two main risk factors for PJI can be divided into patient-related and surgical-related factors. The patient-related factors include gender, age, obesity, systemic diseases like diabetes, smoking, hypoalbuminemia, and preoperative intra-articular injection for TKA [19–25]. The surgical-related factors, on the other hand, include the side of the operation, duration of anesthesia, patella resurfacing, and blood transfusion [26–28]. Medical technology has made managing PJI easier, but reducing its incidence rate still requires significant work.

Gender is a significant factor that affects the incidence of PJI, as per previous research [29–31]. The occurrence of PJI is higher in males (1.29%) than in females (0.67%). The reasons behind this discrepancy could be due to men being more active and aggressive post-TKA, as well as gender-based differences in pharmacokinetics [31–33]. There is not enough research available on the gender-based incidence of the disease, and more observations are needed to establish the same.

The economic and welfare standards of different nations or regions may have an impact on their medical standards [34]. In a recent study, it was found that among the four groups analyzed - Asia, North America, Oceania, and Europe - Asia had a significantly higher incidence of PJI. This could be attributed to various factors such as surgical standards, sterility conditions during operations, and people’s conceptual awareness in underdeveloped regions. These factors may all contribute to the higher occurrence of PJI in Asia [34, 35].Although improving medical standards in underdeveloped areas is a challenging task, we are confident that advancements in time will eventually enhance them.

The incidence and duration of an infection are crucial factors that help in formulating a treatment plan. PJI can be identified within 90 days after surgery, but in some cases, they can be diagnosed as late as six months up to a year later [36–38]. Our research shows a difference between the short-term incidence rate of PJI, which is 1.11%, and its later incidence rate, which is 0.41%. Although the incidence of PJI reduces over an extended period, it is still recommended to monitor patients for an extended period to avoid potential repercussions caused by such infections.

Statistics on the incidence of PJI are often based on clinical studies of a single institution or various databases. In this regard, we conducted a subgroup analysis using data from both sources. Our findings indicate that the incidence of PJI from a single institution is notably higher (1.09%) than that from databases (0.59%). This aligns with the reported incidence of PJI after total hip arthroplasty [15]. However, relying solely on databases for incidence data may lead to underestimation of the actual incidence of PJI.

### Bibliometrics

As a branch of information science, bibliometrics plays an increasingly vital role in scientific domains, especially within the field of medicine. Through bibliometrics, we are able to discern the overall trends, hotspots, and directions of research in a short span of time. Additionally, it provides a visual and simplified structure of knowledge, affording significant convenience to research processes [39, 40]. Web of Science (WoS) is the world’s largest and most comprehensive academic resource hub, encompassing fields such as biomedicine and natural science, and boasting a great number of core journals [41]. The literature data in our study is sourced from this platform. Beyond that, WoS also contains cited references, authors, sources, and publication years. In summary, it offers rapid search capability, advanced search, and citation search, all of which assist us in efficiently and accurately locating desired articles and journal information.

### Global research status

An analysis of literature related to PJI after TKA from 1994 to 2023 reveals that an average of 127.7 papers per year has been published on this subject. In 2011, the number of global publications reached 100 for the first time. This milestone was upstaged in 2016 when the count exceeded 200, reaching over 300 in 2019, and surpassing 400 in 2021. This surge possibly correlates with an increase in TKA numbers due to growing trends of an aging population, and a rise in the incidence of PJI [42, 43]. Despite the growing number of publications, the research intensity in this area remains relatively low compared to other domains, and there is a lack of high-level research reports.

The quantity of articles published within a specific research field can, to a certain extent, appraise the research competence of a country or institution in that area [44]. When looking at the involved nations, the United States far outpaces all others in terms of the volume of publications, markedly surpassing Germany and China, which are ranked second and third, respectively.Seven out of the top ten organizations based on highest publication volume are from the United States, while the remaining three are from Germany. The ‘Journal of Arthroplasty’ has published the highest number of studies related to PJI following TKA. The number of publications in this field is almost twice that of the second and third most published journals - ‘Clinical Orthopaedics and Related Research’ and ‘Journal of Bone and Joint Surgery American Volume’. However, citation analysis indicates a different ranking order for these journals, which is likely due to the fact that significant studies in this field are primarily published in ‘The New England Journal of Medicine’ and ‘Clinical Infectious Diseases’, rather than in the ‘Journal of Arthroplasty’. High-quality clinical studies often require multicenter and large samples, and while there is some collaboration among researchers in various countries, further collaboration is needed to advance the field.

The co-occurrence network map, created by analyzing frequently appearing keywords in the publications included in this study, reveals four clusters of research hotspots related to PJI after TKA. These clusters include risk factors, management, diagnosis, and revision. Several salient themes have been recognized within these clusters, which bear higher weight and larger co-occurrence link strength. For example, the replacement branch mirrors the management of failures post the initial TKA and revision surgeries. The research on PJI after TKA primarily focuses on risk factors, diagnosis, and treatment. Future studies could pivot on these directions, with the aim to provide references and guidance for the prevention and treatment of PJI after TKA. For instance, the search for new serum markers with high specificity to enhance diagnosis efficiency, or the renewal of perioperative management methods to reduce the risks of PJI.

### Limitations

This study has several limitations: First, Our analysis may have gaps in search coverage, but our funnel plot analysis shows minimal impact on results due to limited studies in some subgroups. Second, Although we recognize the differences in methodology and outcomes between database-based studies and clinical research, the high heterogeneity of database-based studies limits our ability to obtain a homogeneous estimate of PJI incidence rate. Third, the collected and screened article data are all sourced from the Web of Science Core Collection, inevitably leading to data omission.This study includes only English-language articles, potentially causing selection bias. Lastly, but equally significant, the Web of Science Core Collection is constantly updated, rendering our analysis results in this study time-constrained. Despite these limitations, our findings still pave the way for guiding future research.

## Conclusion

Our research has found that the total incidence rate of PJI stands at 0.83%. However, when we analyzed different sources of data, we found a significant difference in the incidence of PJI between database sources and individual institutions. The former showed an incidence of only 1.09%, while the latter recorded an incidence rate of 0.59%. Bibliometric studies analyzing the occurrence of PJI after TKA have shown a yearly increase in the number of publications in this field. The United States is the leading country in terms of the quantity of published papers, and is home to institutions that have made the biggest scientific impact. The most influential publication in this area is a literature review by Professor Zimmerli in 2004, which provides a comprehensive summary of the pathogenesis, diagnosis, and treatment of PJI.

## Electronic supplementary material

Below is the link to the electronic supplementary material.

Supplementary Material 1

## Data Availability

Publicly available datasets were analyzed in this study. This data can be found here: www.cdc.gov/nchs/nhanes.
