# Supplementary material for: Incidence of periprosthetic joint infection after primary total knee arthroplasty shows significant variation : a synthesis of meta-analysis and bibliometric analysis
Source: J Orthop Surg Res. 2024 Oct 12;19:649. doi: 10.1186/s13018-024-05099-8 (PMC11470562; doi:10.1186/s13018-024-05099-8)
Supplement: Supplementary file 1 — Supplementary Material 1 [file 13018_2024_5099_MOESM1_ESM.docx]

Supplementary Material

# **Incidence of periprosthetic joint infection after primary total knee arthroplasty is underestimated: a synthesis of meta-analysis and bibliometric analysis**

**Supplementary**

**Figures**


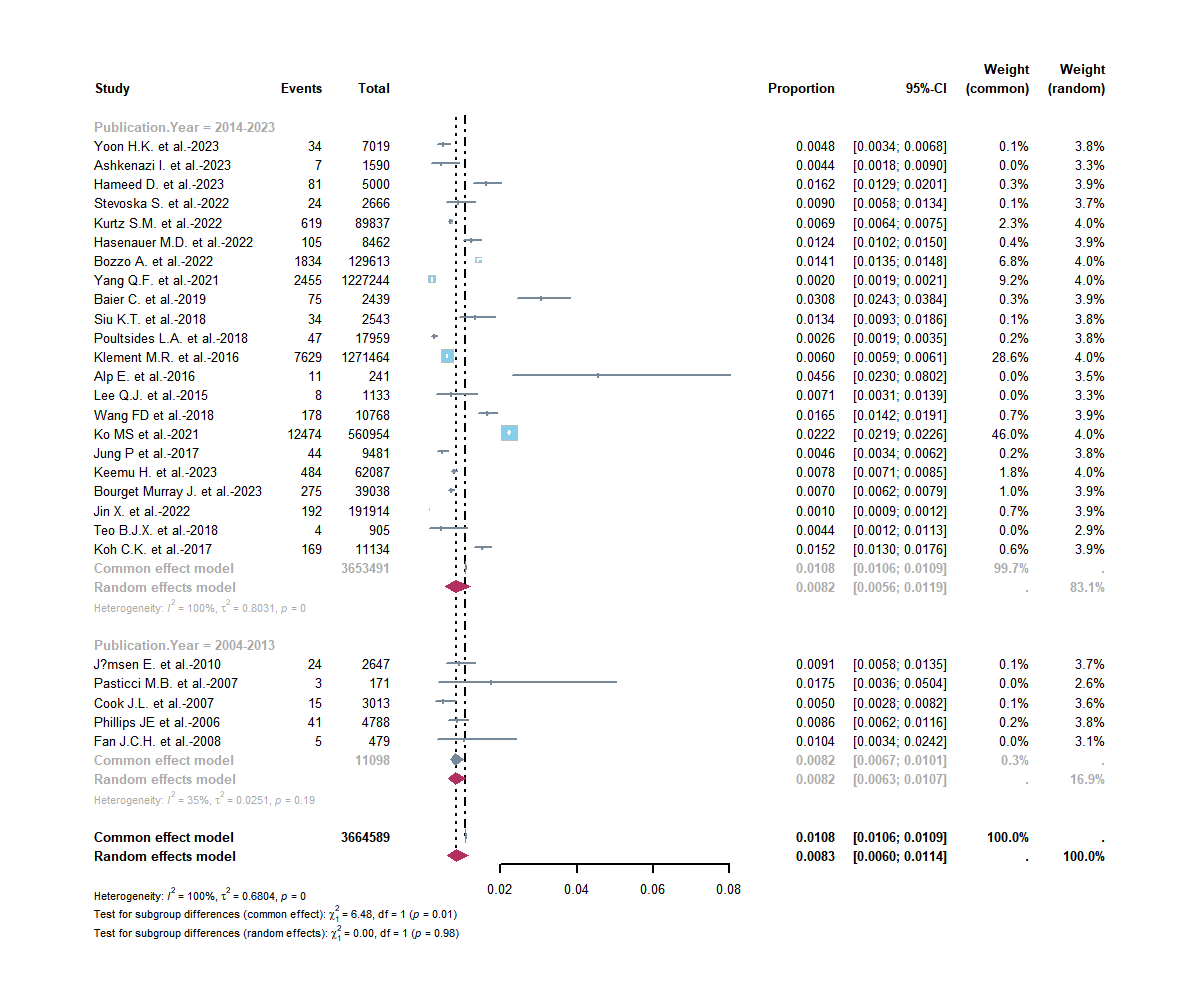


**Supplementary Figure 1. Forest plot of incidence of PJI by publication time.**


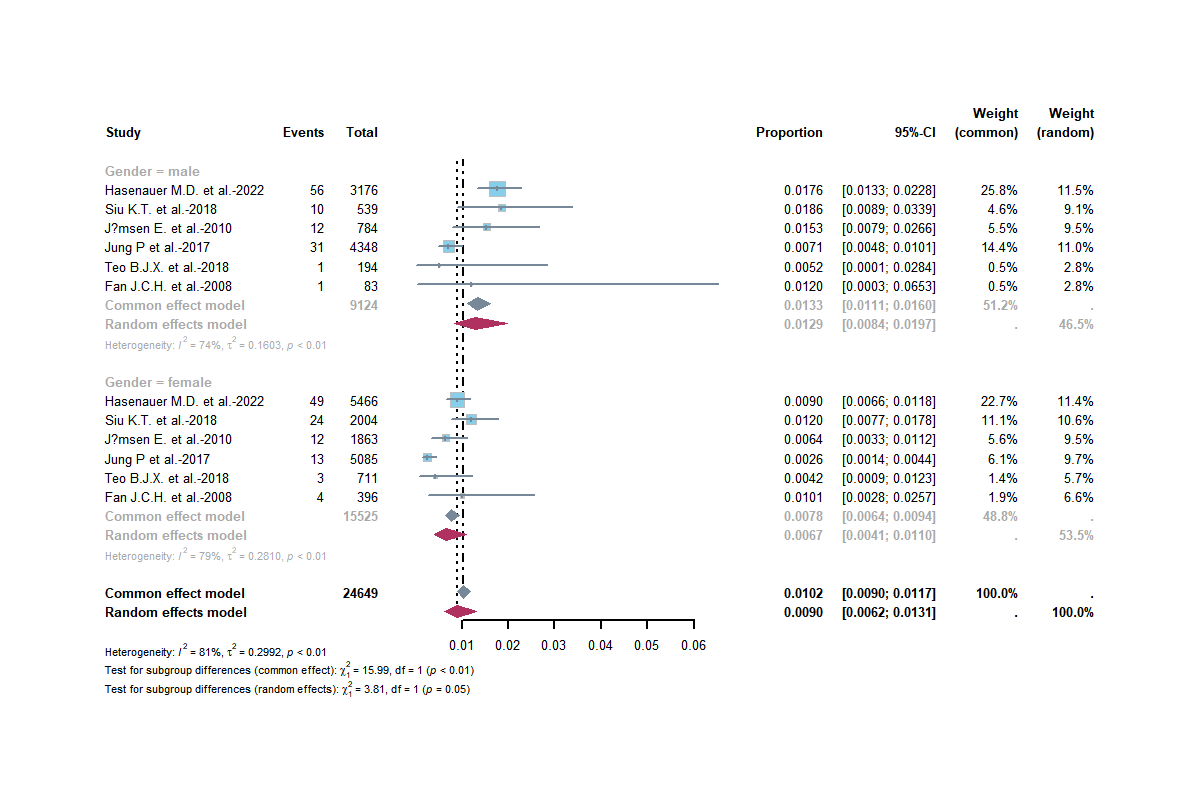


**Supplementary Figure 2. Forest plot of incidence of PJI by by sex**


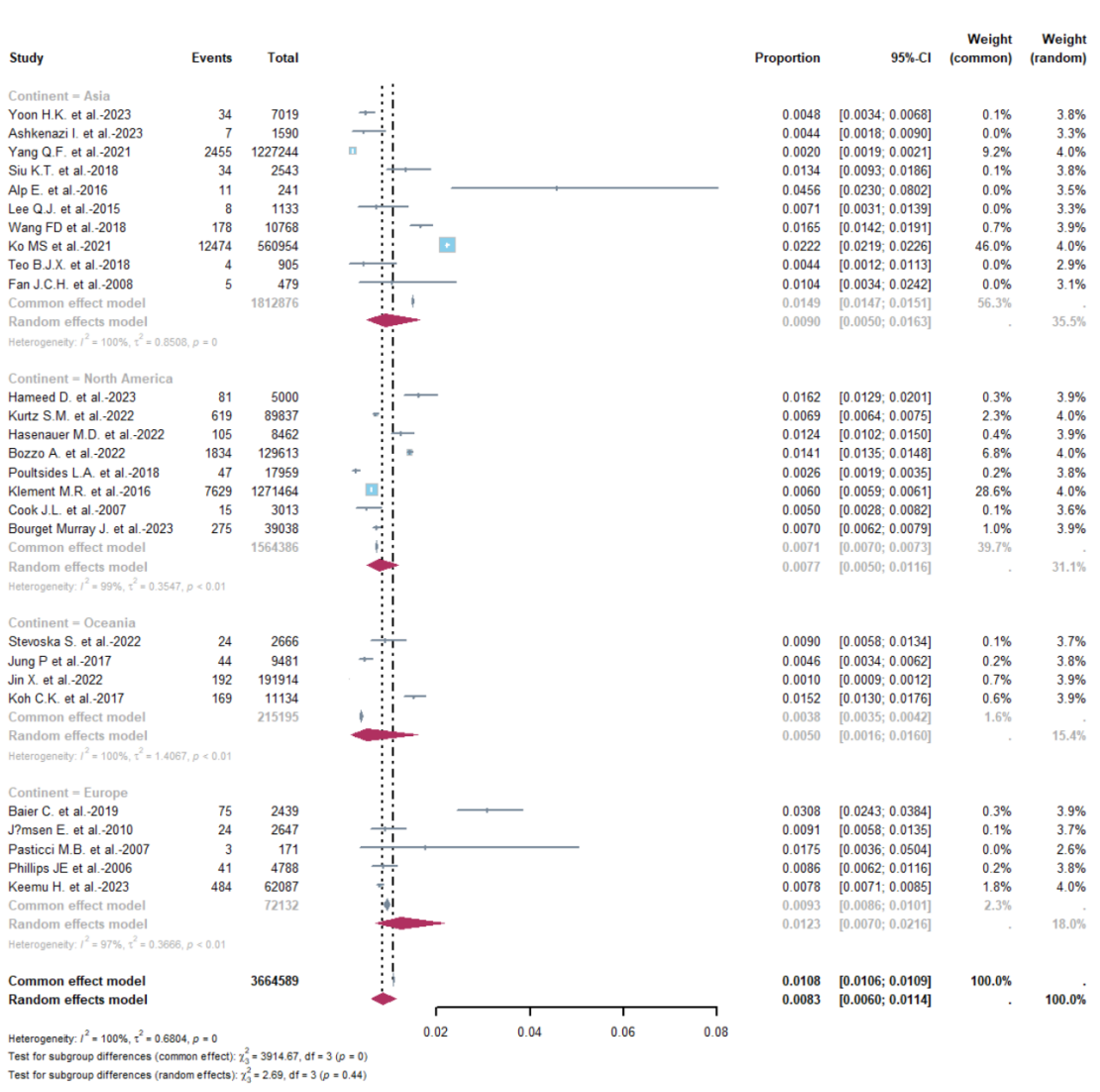


**Supplementary Figure 3. Forest plot of incidence of PJI by by continent**


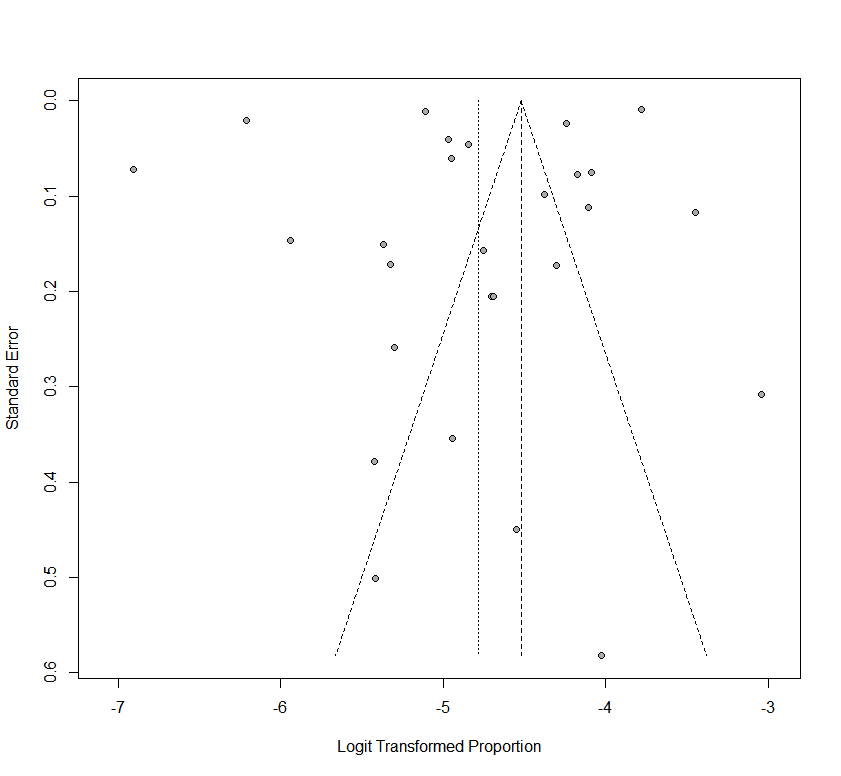


**Supplementary Figure 4 Publication bias of the incidence studies of PJI**


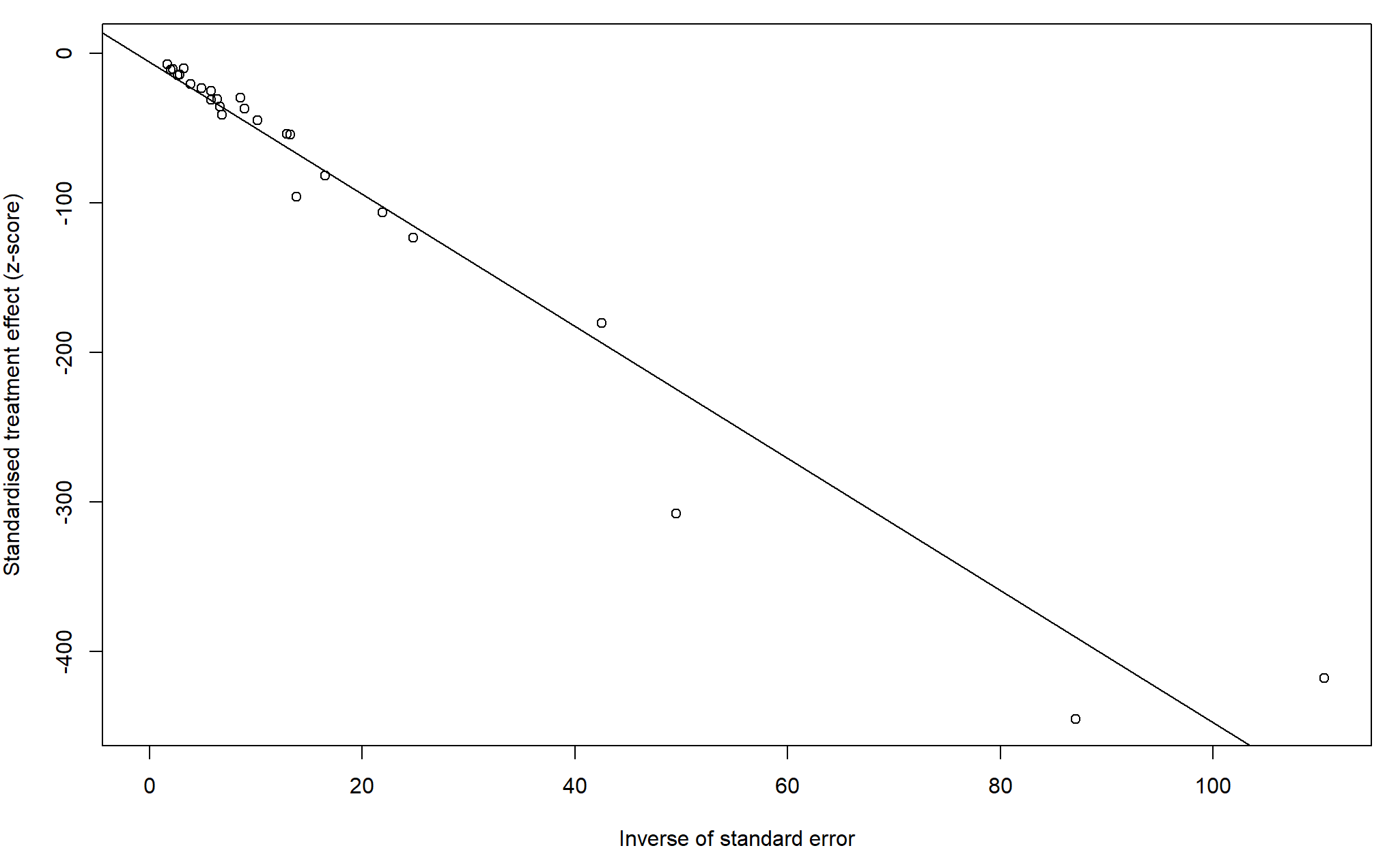
**Supplementary Figure 5 Publication bias of the incidence studies of PJI**


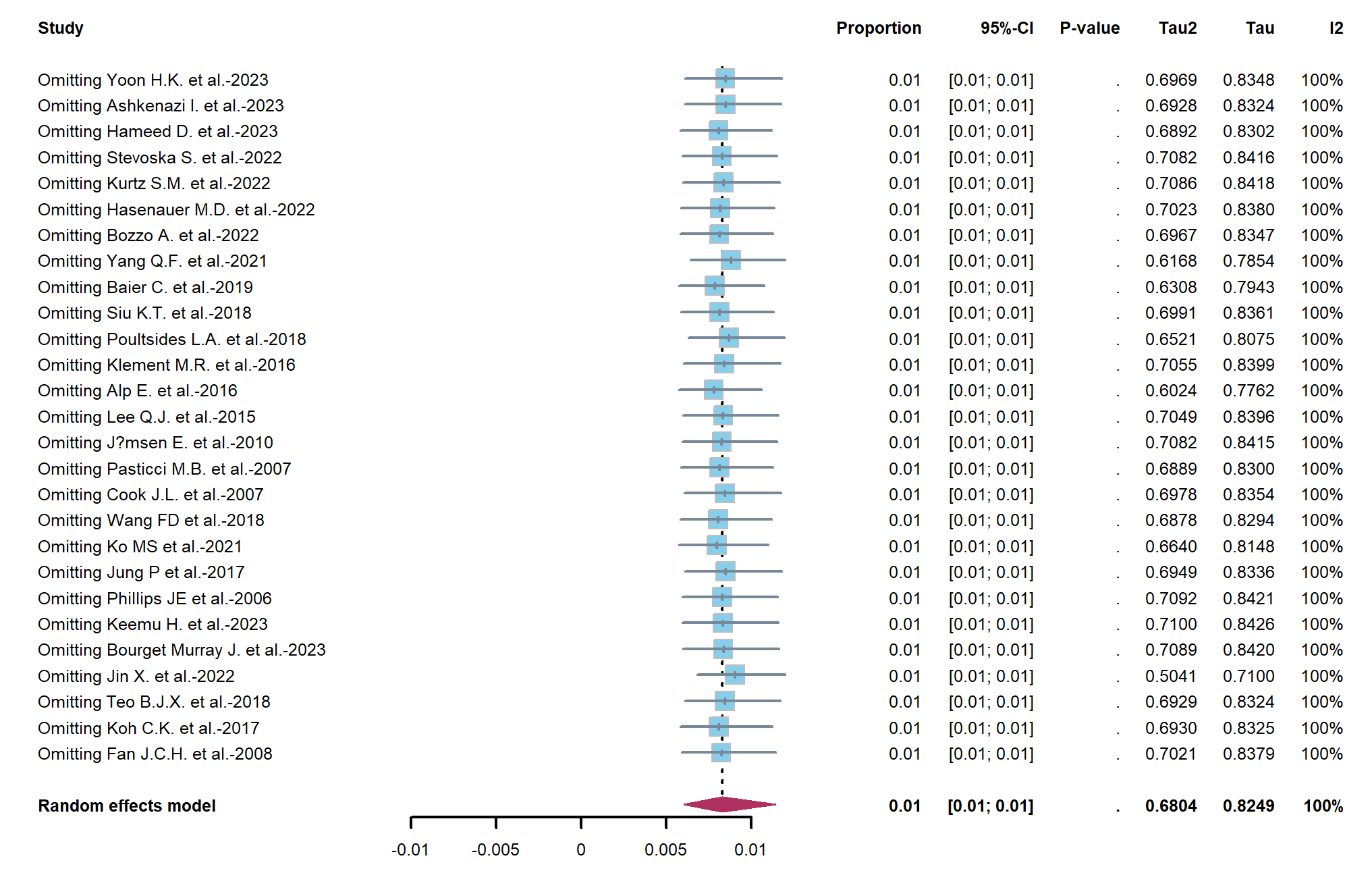


**Supplementary Figure 6 Sensitivity Analysis of the incidence studies of PJI**

**Tables**

| Author,year | Q1 | Q2 | Q3 | Q4 | Q5 | Q6 | Q7 | Q8 | Q9 | Total(%) | Risk of Bias |
| --- | --- | --- | --- | --- | --- | --- | --- | --- | --- | --- | --- |
| Hasenauer M.D. et al. 2022 | N | Y | Y | Y | Y | Y | U | Y | Y | 77.78 | Low |
| Siu K.T. et al. 2018 | N | Y | Y | Y | Y | Y | U | Y | Y | 77.78 | Low |
| Jämsen E. et al. 2010 | N | Y | Y | Y | Y | Y | Y | Y | U | 77.78 | Low |
| Jung P et al. 2017 | Y | Y | Y | Y | Y | Y | U | Y | Y | 88.89 | Low |
| Teo B.J.X. et al. 2018 | N | Y | Y | Y | Y | Y | Y | Y | U | 77.87 | Low |
| Fan J.C.H. et al. 2008 | N | Y | Y | U | Y | Y | Y | Y | Y | 77.78 | Low |
| Yoon H.K. et al. 2023 | Y | Y | Y | Y | N | Y | N | U | U | 55.56 | Moderate |
| Stevoska S. et al. 2022 | N | Y | Y | Y | U | Y | U | Y | U | 55.56 | Moderate |
| Bozzo A. et al.2022 | Y | Y | Y | Y | Y | Y | U | U | U | 66.67 | Moderate |
| Poultsides L.A. et al. 2018 | Y | Y | Y | N | Y | U | N | Y | U | 55.56 | Moderate |
| Klement M.R. et al. 2016 | Y | Y | Y | U | U | Y | U | Y | U | 55.56 | Moderate |
| Lee Q.J. et al. 2015 | Y | Y | Y | Y | U | Y | U | U | Y | 55.56 | Moderate |
| Pasticci M.B. et al. 2007 | N | Y | Y | Y | Y | Y | U | N | U | 55.56 | Moderate |
| Cook J.L. et al. 2007 | Y | Y | Y | U | Y | Y | U | Y | U | 66.67 | Moderate |
| Wang FD et al. 2018 | N | Y | Y | Y | Y | Y | Y | U | U | 66.67 | Moderate |
| Keemu H. et al. 2023 | Y | Y | Y | Y | U | Y | U | Y | U | 66.67 | Moderate |
| Ashkenazi I. et al. 2023 | N | Y | N | Y | Y | N | U | Y | U | 44.45 | High |
| Hameed D. et al. 2023 | Y | Y | Y | Y | N | U | U | U | U | 44.45 | High |
| Kurtz S.M. et al. 2022 | Y | Y | Y | U | U | Y | U | N | U | 44.45 | High |
| Yang Q.F. et al. 2021 | Y | Y | U | Y | U | Y | U | U | N | 44.45 | High |
| Baier C. et al. 2019 | N | Y | Y | U | Y | Y | U | N | U | 44.45 | High |
| Alp E. et al. 2016 | N | Y | Y | Y | U | Y | U | U | N | 44.45 | High |
| Ko MS et al. 2021 | Y | Y | Y | N | U | N | U | Y | U | 44.45 | High |
| Phillips JE et al. 2006 | N | Y | Y | Y | U | U | Y | U | U | 44.45 | High |
| Bourget Murray J. et al. 2023 | Y | Y | Y | U | U | Y | N | U | U | 44.45 | High |
| Jin X. et al. 2022 | Y | Y | Y | U | U | Y | U | U | N | 44.45 | High |
| Koh C.K. et al. 2017 | N | Y | Y | Y | U | Y | U | U | U | 44.45 | High |
| Legend:Y=Yes;N=No;U=Unclear;NA=Not applicable | | | | | | | | | | | |
| Risk of bias was categorized as high when the study reaches up to 49% score "yes" ,moderate wthen the study reached 50% to 69% | | | | | | | | | | | |
| score "yes" ,and low when the study reached more than 70% score "yes". | | | | | | | | | | | |

**Supplementary Table 1 the assessment scores of the articles**

| 1994 | 5 |
| --- | --- |
| 1995 | 7 |
| 1996 | 9 |
| 1997 | 10 |
| 1998 | 9 |
| 1999 | 12 |
| 2000 | 7 |
| 2001 | 15 |
| 2002 | 15 |
| 2003 | 19 |
| 2004 | 15 |
| 2005 | 25 |
| 2006 | 46 |
| 2007 | 38 |
| 2008 | 40 |
| 2009 | 53 |
| 2010 | 68 |
| 2011 | 84 |
| 2012 | 106 |
| 2013 | 110 |
| 2014 | 164 |
| 2015 | 180 |
| 2016 | 216 |
| 2017 | 250 |
| 2018 | 299 |
| 2019 | 340 |
| 2020 | 365 |
| 2021 | 407 |
| 2022 | 424 |
| 2023 | 425 |

**Supplementary Table 2 Number of articles published per year**

| 机构名称 | 所在国家或地区 | 发文量 |
| --- | --- | --- |
| ROTHMAN INSTITUTE | 美国 | 271 |
| JEFFERSON UNIVERSITY | 美国 | 247 |
| MAYO CLINIC | 美国 | 220 |
| CLEVELAND CLINIC FOUNDATION | 美国 | 117 |
| HARVARD UNIVERSITY | 美国 | 117 |
| FREE UNIVERSITY OF BERLIN | 德国 | 107 |
| HUMBOLDT UNIVERSITY OF BERLIN | 德国 | 105 |
| CHARITE UNIVERSITATSMEDIZIN BERLIN | 德国 | 104 |
| RUSH UNIVERSITY | 美国 | 100 |
| HOSP SPECIAL SURG | 美国 | 98 |

**Supplementary Table 3 Analysis of the most productive institutions**
